# Beneficial effects of proprietary Chinese medicines as adjuvant therapy in patients with advanced colorectal cancer: A meta-analysis

**DOI:** 10.1097/MD.0000000000043147

**Published:** 2025-08-01

**Authors:** Mingxing Wang, Wanhui Dong, Qingming Sun, Gongyi Wu

**Affiliations:** a Department of Medical Oncology, Lu’an Hospital of Traditional Chinese Medicine Affiliated to Anhui University of Chinese Medicine, Lu’an, Anhui Province, China; b Department of Orthopedics, Lu’an Hospital of Traditional Chinese Medicine Affiliated to Anhui University of Chinese Medicine, Lu’an, Anhui Province, China.

**Keywords:** Huachansu capsules, mesh meta-analysis, mid to advanced stage colorectal cancer, randomized controlled trials, traditional Chinese patent medicine, Yangzheng Xiaoji capsules

## Abstract

**Background:**

In the clinical management of advanced colorectal cancer, traditional Chinese patent medicines or their simple preparations are commonly combined with conventional chemotherapy; however, robust comparative evidence on efficacy and safety across regimens is lacking. Because the submission system does not allow relabeling the section heading, we have clarified this change here.

**Methods:**

Randomized controlled trials investigating the use of traditional Chinese patent medicines and simple preparations in combination with conventional chemotherapy for the treatment of advanced colorectal cancer were searched in the following databases: CNKI, Wanfang, VIP, PubMed, Cochrane Library, Embase, and Web of Science. The search was conducted from the establishment of the databases until October 20, 2023, using computer-based searches. Two individuals independently screened, extracted data, and assessed the quality of the literature. Used RevMan and Stata 16.0 software to analyze the data.

**Results:**

A total of 45 studies were included, involving 3888 patients with advanced colorectal cancer. The intervention measures in the experimental group included Huachansu capsules, Brucea Javanica Oil Emulsion injection, Kanglaite, Xiaoaiping, compound cantharidin capsules, Kangai injection, Yangzheng Xiaoji capsules, Tenglong Buzhong Tang, Aidi injection, and Compound Sophora flavescens injection, in combination with conventional chemotherapy. The control group was treated with conventional chemotherapy. The mesh meta-analysis results showed that the combination of conventional chemotherapy has the following benefits for colorectal cancer patients: Yangzheng Xiaoji capsule is the best in terms of improving the objective response rate (ORR), disease control rate (DCR), quality of life (QOL), and immune function. Huachansu capsule is the best in terms of reducing the decline of white blood cell count and the incidence of nausea and vomiting after chemotherapy.

**Conclusion:**

The existing evidence indicates that the combination of 10 traditional Chinese patent medicines and simple preparations, in addition to conventional chemotherapy, can improve the DCR, ORR, and QOL for patients with advanced colorectal cancer. Furthermore, this combination treatment can improve patients’ immune function and reduce the adverse reactions associated with chemotherapy. Among them, Yangzheng Xiaoji capsules have the best effect in improving immune function, ORR, DCR, and QOL, Huachansu capsules have the best effect in improving white blood cell count decline and reducing nausea and vomiting after chemotherapy.

## 1. Introduction

With the change of lifestyle, the incidence rate of colorectal cancer (CRC) is increasing year by year.^[1]^ Colorectal cancer, including colon cancer and rectal cancer, ranks fifth in the incidence of malignant tumors, and its incidence rate accounts for 10%~15% of all tumors.^[2]^ Chemotherapy is one of the main treatment methods for colorectal cancer at present.^[3]^ With the continuous increase in the incidence rate of colorectal cancer, the research on the clinical treatment of colorectal cancer is becoming more and more diversified. At present, the first recognized treatment method for colorectal cancer is still western medicine, mainly including surgery, radiotherapy and chemotherapy, and targeted treatment. However, the complications, recurrence rate, and adverse reactions after radiotherapy and chemotherapy of patients after surgery seriously affect the quality of life (QOL) of patients; With the continuous development of China’s traditional Chinese medicine (TCM) industry and the accumulation of clinical experience, the clinical advantages of TCM in preventing and treating colorectal cancer, especially in intervening in adverse reactions after chemotherapy for colorectal cancer, continue to highlight.^[4,5]^ Therefore, seeking efficient and low-toxicity combination drugs against colorectal cancer has always been a hot topic in clinical research.

Chinese medicine has made certain achievements in the prevention and treatment of colorectal cancer, and many studies have shown that traditional Chinese patent medicines and simple preparations can reduce the toxicity of chemotherapy and improve the antitumor efficacy and the QOL of patients.^[6,7]^ At present, many studies have reported that traditional Chinese patent medicines and simple preparations combined with chemotherapy have a good effect in the treatment of colorectal cancer, but due to the lack of comparison between the 2, it is still unclear which traditional Chinese patent medicines and simple preparations are more advantageous. The biggest advantage of mesh meta-analysis is that it can quantitatively compare different intervention measures for treating similar diseases and sort them according to a certain outcome indicator to select the best intervention measures. Therefore, this study used the mesh meta-analysis method to evaluate the efficacy and safety of traditional Chinese patent medicines and simple preparations combined with chemotherapy in the treatment of colorectal cancer, in order to provide evidence-based evidence for clinical use.

## 2. Methods

Under the ID CRD42024520339, the study protocol was uploaded to the International Prospective Register of Systematic Reviews database.

### 2.1. Literature search

A computer search was conducted on PubMed, Cochrane Library, Embase, Web of Science, Wanfang Database, VIP Database, and CNKI, which are the 7 major databases. The retrieval time spanned from the establishment of each database to October 20, 2023. The search is conducted by combining keywords with additional terms, and adjustments are made based on the specific features of each database. Chinese search terms include colorectal cancer, colon cancer, rectal cancer, traditional Chinese patent medicines, simple preparations, and randomized controlled studies. The English search terms include Colorectal tumor, Colorectal cancer, Colon tumor, Colon cancer, Rectal tumor, Rectal cancer, Chinese patient medicine, randomized controlled study, etc. Take the PubMed database as an example, the search formula is as follows: ((((((((((((((((Colorectal Neoplasms[MeSH Terms]) OR (Colorectal Neoplasm[Title/Abstract])) OR (Neoplasm, Colorectal[Title/Abstract])) OR (Colorectal Tumors[Title/Abstract])) OR (Colorectal Tumor[Title/Abstract])) OR (Tumor, Colorectal[Title/Abstract])) OR (Tumors, Colorectal[Title/Abstract])) OR (Neoplasms, Colorectal[Title/Abstract])) OR (Colorectal Cancer[Title/Abstract])) OR (Cancer, Colorectal[Title/Abstract])) OR (Cancers, Colorectal[Title/Abstract])) OR (Colorectal Cancers[Title/Abstract])) OR (Colorectal Carcinoma[Title/Abstract])) OR (Carcinoma, Colorectal[Title/Abstract])) OR (Carcinomas, Colorectal[Title/Abstract])) OR (Colorectal Carcinomas[Title/Abstract])) AND (((((((((((((((((((((((((Medicine, Chinese Traditional[MeSH Terms]) OR (Zhong Yi Xue[Title/Abstract])) OR (Chung I Hsueh[Title/Abstract])) OR (Hsueh, Chung I[Title/Abstract])) OR (Traditional Medicine, Chinese[Title/Abstract])) OR (Chinese Traditional Medicine[Title/Abstract])) OR (Traditional Chinese Medicine[Title/Abstract])) OR (Chinese Medicine, Traditional[Title/Abstract])) OR (Traditional Tongue Diagnosis[Title/Abstract])) OR (Tongue Diagnoses, Traditional[Title/Abstract])) OR (Tongue Diagnosis, Traditional[Title/Abstract])) OR (Traditional Tongue Diagnoses[Title/Abstract])) OR (Traditional Tongue Assessment[Title/Abstract])) OR (Tongue Assessment, Traditional[Title/Abstract])) OR (Traditional Tongue Assessments[Title/Abstract])) OR (Huachansu Capsules[Title/Abstract])) OR (Brucea Javanica Oil Emulsion Injection[Title/Abstract])) OR (Kanglaite[Title/Abstract])) OR (Xiaoaiping[Title/Abstract])) OR (Compound Cantharidin Capsules[Title/Abstract])) OR (Kangai Injection[Title/Abstract])) OR (Yangzheng Xiaoji Capsules[Title/Abstract])) OR (Tenglong Buzhong Tang[Title/Abstract])) OR (Aidi Injection[Title/Abstract])) OR (Compound Sophora flavescens Injection[Title/Abstract]))

### 2.2. Inclusion and exclusion criteria

#### 2.2.1. Inclusion criteria

Research type: all included studies are randomized controlled trials (RCTs) on traditional Chinese patent medicines and simple preparations combined with chemotherapy for colorectal cancer. Research subjects: confirmed as colorectal cancer by histopathology or cytology, with unlimited pathological cell typing and no age, gender, race, or etiology limitations. Intervention measures: the experimental group received traditional Chinese patent medicines and simple preparations combined with conventional chemotherapy. The control group consisted of conventional chemotherapy regimens such as capecitabine + oxaliplatin regimen (oxaliplatin + capecitabine), FOLFOX regimen (5-fluorouracil + oxaliplatin + calcium folinate), Capecitabine, Tegafur, PT regimen (paclitaxel + cisplatin), CapeOX regimen (carboplatin + pemetrexed), and FOLFIRI regimen (5-fluorouracil + calcium folinate + irinotecan + cisplatin). Outcome measures: objective response rate (ORR), disease control rate (DCR), QOL, immune function (CD4+/CD8+), white blood cell count (WBC), nausea and vomiting.

#### 2.2.2. Exclusion criteria

Research types such as reviews and animal experiments. Literature and repeated publications or conference abstracts other than Chinese and English. Patients with a pathologically confirmed diagnosis of noncolorectal cancer. Literature with inconsistent outcome indicators. Intervention measures combined with radiotherapy, chemotherapy, and other TCM literature. Literature with obvious data errors. Traditional Chinese patent medicines and simple preparation varieties with <2 articles.

### 2.3. Literature screening and data extraction

After importing the retrieved literature into EndNote X9 literature management software (Clarivate Analytics, Philadelphia) for deduplication, 2 researchers independently screened the literature, extracted the data, and cross-checked it. If there are any differences, they can be resolved through discussion or consultation with a third party. When selecting literature, first read the title, and after excluding significantly unrelated literature, further read the abstract and full text to determine whether to include it. The content of data extraction includes: basic information included in the study, such as research topic, first author, published journal, etc. Baseline characteristics and intervention measures of the study subjects. Key elements of bias risk assessment. Outcome indicators and outcome measurement data of concern.

### 2.4. Risk assessment of bias included in the study

Two researchers independently evaluated the risk of bias included in the study and cross-checked the results. The bias risk assessment was conducted using the RCT bias risk assessment tool recommended in Cochrane Manual 5.1.0 (The Cochrane Collaboration, Oxford, England, United Kingdom).

### 2.5. Statistical analysis

Using Review Manager 5.3 (The Cochrane Collaboration, Oxford, England, United Kingdom) to draw a risk bias map, a network meta-analysis of the random effects model was conducted using Stata 16.0 software (StataCorp LLC, College Station) within the frequency domain framework. The results of binary and continuous variables were represented by odds ratio (OR) and mean difference values, as well as their respective 95% confidence interval (CI). When there is a closed loop, inconsistency testing is required. However, since there is no closed loop among the intervention measures included in this study, which means that all pairwise comparisons between intervention drugs come from indirect comparisons, inconsistency testing is not necessary. The results can be directly analyzed statistically under a consistent model. Use the network group command to draw evidence network diagrams, comparison correction funnels, pairwise comparison forest diagrams, league tables, and surface under the cumulative ranking (SUCRA) diagrams. The results of the network meta-analysis are presented in the league table, where the 95% CI of the binary variable OR does not cross 1 or the 95% CI of the continuous variable mean difference does not cross 0, indicating a statistically significant difference; present evidence network relationships using evidence network diagrams; present publication bias and small sample effects in a funnel plot; present the best possible intervention measures using a probability ranking chart; and use the SUCRA to rank each indicator for each intervention measure. The larger the area under the curve, the higher the ranking, indicating that the intervention measure is better. By utilizing a comparison adjusted funnel plot to identify potential publication bias in small and large research findings (typically involving a minimum of 10 studies), the approach involves calculating the disparity between the effects of a paired comparison in each study and the combined effects of all similar comparisons. Subsequently, a regression analysis is performed on the calculated disparity using the standard error of the effects. This results in the addition of a straightforward linear regression line to the funnel plot, which aids in a more intuitive assessment of the extent of bias in the research outcomes.

## 3. Results

### 3.1. Literature search results

**Figure 1.**
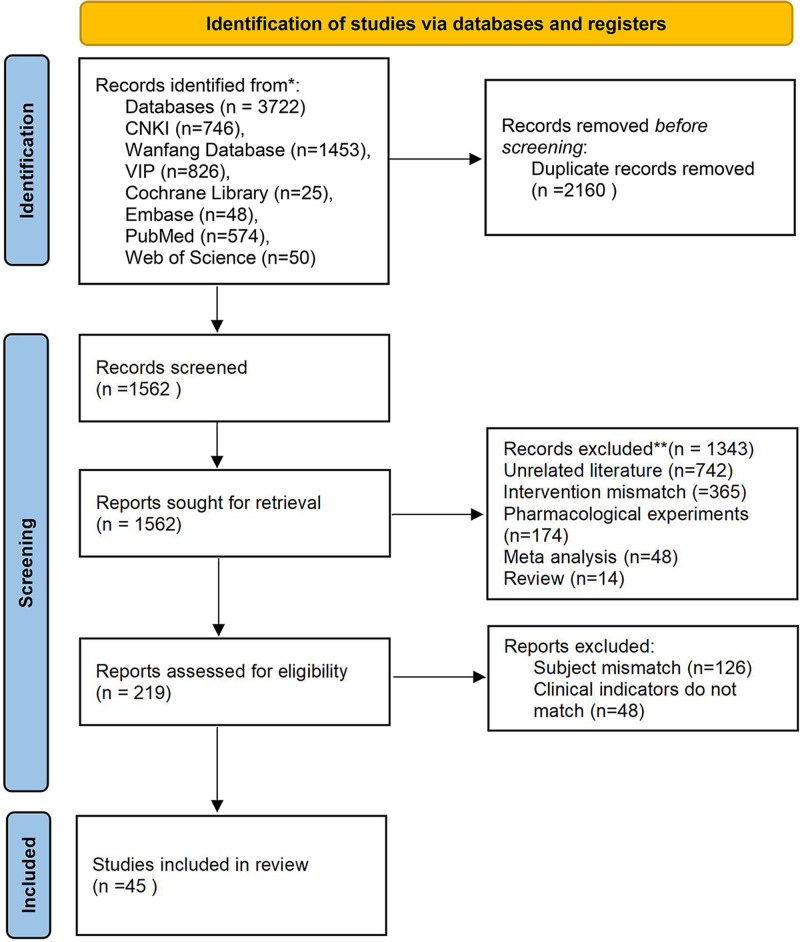
Literature screening process and rests.

According to the pre-established retrieval strategy, a total of 3722 relevant literature were obtained during the initial examination. Layer-by-layer screening was conducted according to the established inclusion and exclusion criteria, and 45 studies^[4,8–51]^ were ultimately included, all of which were Chinese literature. The literature screening process and results are shown in Figure 1. A total of 3888 patients were included in the 45 RCTs, published from 2011 to 2022, all of which were double-arm trials. There are 10 kinds of traditional Chinese patent medicines and simple preparations involved, including 6 RCTs of Huachansu capsule, 6 RCTs of Brucea javanica oil emulsion injection, 4 RCTs of Kanglaite, 5 RCTs of Xiaoaiping, 5 RCTs of compound cantharides capsule, 4 RCTs of Kangai injection, 4 RCTs of Yangzheng Xiaoji capsule, 1 RCT of Tenglong Buzhong decoction, 5 RCTs of Aidi injection, and 5 RCTs of compound sophora flavescens injection. The key features of the literature included are presented in Table 1.

**Table 1:** Basic characteristics of included literature.

Reference (year)	Gender (male/female)		Stage	Cancer type	Age (years)		Treatment		Dosage	Usage cycle	Clinical outcomes
	T	C			T	C	T	C			
Zhanguo^[8]^	15/9	14/10	Advanced	Colon	55.82 ± 8.04	55.09 ± 7.26	HCS + FOLFOX4	FOLFOX4	0.5 g Tid	(1–21 days) × 4	①②③④⑤
Miao^[9]^	19/15	17/17	Advanced	Colon	35.34 ± 4.67	35.67 ± 2.31	HCS + FOLFOX4	FOLFOX4	0.75 g Bid	(1–21 days) × 4	①②③④⑤
Jinpeng et al^[10]^	24/24	22/26	Advanced	Colon	58.2	58.7	HCS + XELOX	XELOX	0.5 g Tid	(1–21 days) × 2	①②③④⑤
Yan-jiang et al^[11]^	16/18	17/17	Ⅲ	Colon	35.57 ± 2.35	35.42 ± 4.78	HCS + FOLFOX4	FOLFOX4	0.6 g Tid	(1–21 days) × 4	①②③④⑤
Hui et al^[12]^	21/14		Unclear	Colorectal	56		HCS + FOLFOX6	FOLFOX6	0.9 g Tid	(1–21d)*4	①②③④
Ming-wei^[13]^	30/31	32/29	Unclear	Colorectal	56.8 ± 5.8	55.1 ± 6.4	HCS + XELOX	XELOX	0.75 g Bid	(1–21 days) × 6	④⑤
Youwei^[14]^	45/45	43/47	Advanced	Colon	45.32 ± 4.88	45.27 ± 4.47	YDZ + capecitabine	Capecitabine	30 mL Qd	(1–7 days) × 8	①②⑤
Ting-ting et al^[15]^	24	12	Advanced	Colon	51–76	53–77	YDZ + capecitabine	Capecitabine	30 mL Qd	(1–7 days) × 8	①②④⑤
Hongxia^[16]^	20/10	21/9	Ⅲ–Ⅳ	Colon	50	52	YDZ + FOLFOX	FOLFOX	30 mL Qd	(1–7 days) × 3	①②③④⑤
Jing^[17]^	21/13	20/14	Advanced	Colorectal	57.88 ± 4.73	57.24 ± 5.12	YDZ + FOLFOX	FOLFOX	30 mL Qd	unclear	①②③④⑤⑥
Mengyan et al^[18]^	63/37		Unclear	Colorectal	54.89 ± 12.34		YDZ + FOLFOX	FOLFOX	30 mL Qd	(1–30d)*3	①②⑤
Qiyun et al^[19]^	12/8	13/8	Ⅳ	Colorectal	56	57	YDZ + FOLFOX6	FOLFOX6	20 mL Qd	(1–7 days) × 2	①②③
Wen-qun^[20]^	26/20	25/21	Ⅲ	Colon	47.60 ± 6.39	46.87 ± 5.91	KLT + XELOX	XELOX	100 mL Qd	(1–7 days) × 4	①②⑤⑥
Tao^[21]^	16/9		Ⅲ–Ⅳ	Colorectal	>60		KLT + Tegafur	Tegafur	200 mL Qd	(1–21d)*2	①②⑤
Xiaoda and Da^[22]^	19/14	18/14	Ⅳ	Colorectal	67		KLT + CapeOX	CapeOX	100 mL Qd	(1–14d)*2	①②③④⑤⑥
Lu and Qiumin^[23]^	22/14	23/13	Ⅳ	Colorectal	58.3 ± 4.9	57.6 ± 4.2	KLT + Tegafur	Tegafur	200 mL Qd	(1–7 days) × 6	①②⑤
Ke and Wei^[24]^	11/12	9/14	Ⅱ–Ⅳ	Colon	52. 1 ± 14. 8	52.7 ± 12.9	XAP + TP	TP	3 g Bid	(1–21 days) × 3	①②③④⑤⑥
Pei and Jialin^[25]^	31/27	30/28	Unclear	Colorectal	51.2 ± 8.5	50.1 ± 8.3	XAP + XELOX	XELOX	60 mL Qd	(1–14 days) × 3	①②⑤⑥
Fan et al^[26]^	17/13	16/14	Ⅱ–Ⅲ	Colorectal	52.8 ± 11.2	49.6 ± 5.5	XAP + FOLFOX4	FOLFOX4	30 mL Qd	(1–7 days) × 2	①②⑤
Yantao^[27]^	20/12	30/19	Advanced	Colorectal	Unclear		XAP + XELOX	XELOX	60 mL Qd	(1–14d) × 4	①②③④⑤
Ling^[28]^	12/8	13/7	Advanced	Colon	59.24 ± 20.37	61.56 ± 21.53	XAP + FOLFOX4	FOLFOX4	1 mg Qd	(1–14 days) × 2	①②③④⑤
Youguo et al^[29]^	21/24	18/22	Ⅲ–Ⅳ	Colon	58.73 ± 2.19	58.56 ± 2.07	FFBM + mFOLFOX6	mFOLFOX6	0.75 g Bid	(1–7 days) × 6–24	①②③④⑤⑥
Sumei and Shengwen^[30]^	19/11	18/12	Ⅲ–Ⅳ	Colorectal	59.39 ± 5.65	59.47 ± 5.69	FFBM + Tegafur	Tegafur	0.75 g Bid	(1–30 days) × 3	①②③④⑤⑥
Guang-cai et al^[31]^	31/18	33/16	Ⅳ	Colon	68.2 ± 6.1	67.9 ± 6.2	FFBM + FOLFOX6	FOLFOX6	0.75 g Bid	(1–30 days) × 3	①②③④⑤
Bingxin et al^[4]^	30/16	34/12	Ⅱ–Ⅲ	Colorectal	55.61	57.21	FFBM + FOLFOX4	FOLFOX4	30–50 mL Qd	(1–14 days) × 4	①②
Huijun et al^[32]^	22/28	23/27	Unclear	Rectal	32.68 ± 5.78	32.53 ± 5.52	FFBM + FOLFOX	FOLFOX	0.75 g Bid	(1–30 days) × 3	①②⑥
Qirong et al^[33]^	30/25	28/27	Ⅲ–Ⅳ	Colon	46 ± 4	45 ± 3	KAZS + CapeOX	CapeOX	40 mL Qd	(1–14 days) × 3	①②③⑤
Il-ming et al^[34]^	23/15	26/12	Ⅲ–Ⅳ	Colon	49.57 ± 1.30	49.34 ± 1.37	KAZS + CapeOX	CapeOX	40 mL Qd	(1–14 days) × 2	①②⑤⑥
Hao et al^[35]^	16/10	17/9	Ⅲ-Ⅳ	Colon	45.30 ± 5.32	45.00 ± 4.38	KAZS + CapeOX	CapeOX	40 mL Qd	(1–14 days) × 2	①②③⑤
Bin et al^[36]^	36/32	37/31	ⅡB–Ⅲ	Colon	57.8 ± 10.9	57.4 ± 10.6	KAZS + FOLFOX	FOLFOX	40 mL Qd	(1–10 days) × 4	①②④⑥
Bo and Xueling^[37]^	20/19	13/26	Advanced	Colorectal	49~70	48~66	YZXJ + FOLFOX	FOLFOX	1.56 g Tid	60 days	①②③⑤
Jiahe et al^[38]^	92/68	89/71	Ⅲ–Ⅳ	Colorectal	60.5	62	YZXJ + FOLFOX	FOLFOX	1.56 g Tid	(1–14 days) × 4	①②⑥
Yan and Yanlong^[39]^	44/26	41/29	Ⅳ	Colon	54.65 ± 10.98	54.95 ± 11.09	YZXJ + FOLFOX4	FOLFOX4	1.56 g Tid	(1–21 days) × 8–12	①②③④⑤
Yong-qing et al^[40]^	28/14	29/13	Unclear	Colorectal	60.1 ± 2.2	59.4 ± 2.1	YZXJ + FOLFIRI	FOLFIRI	1.56 g Tid	(1–14 days) × 6	①②⑤
Bing et al^[41]^	32/21	30/22	Advanced	Colorectal	54.3	53.2	TLBZ + CapeOX	CapeOX	Unclear	(1–21 days) × 2	①②⑤
Gang et al^[42]^	39/21	36/24	Advanced	Colorectal	61. 48 ± 5. 25	59. 79 ± 5. 48	AD + FOLFIRI	FOLFIRI	100 mL Qd	(1–14 days) × 4	①②⑤
Youlin et al^[43]^	22/18	20/20	Advanced	Colorectal	64.37 ± 18.03	65.87 ± 17.12	AD + FOLFIRI	FOLFIRI	50–100 mL Qd	Unclear	①②⑤
Xueyuan^[44]^	16/15	17/13	Advanced	Colorectal	63.6 ± 7.5	64.1 ± 8.2	AD + FOLFIRI	FOLFIRI	50–100 mL Qd	(1–14 days) × 2–6	①②⑤
Zhen et al^[45]^	20/11	18/13	Advanced	Colorectal	54.3	55.2	AD + FOLFIRI	FOLFIRI	50 mL Qd	(1–14 days) × 4	①②③④⑤
Mingquan^[46]^	21/13	22/12	Advanced	Colorectal	56.8 ± 3.6	56.4 ± 3.6	AD + FOLFIRI	FOLFIRI	100 mL Qd	14 days	①②⑤
Weizhi and Xu^[47]^	21/18	26/13	Ⅰ–Ⅱ	Colorectal	59.13 ± 4.18	58.25 ± 5.67	FFKS + FOLFOX4	FOLFOX4	20 mL Qd	(1–14 days) × 4	①②④⑤⑥
Zhen-tao^[48]^	28/21	31/18	Ⅱ–Ⅳ	Colorectal	56.17 ± 5.36	56.09 ± 5.18	FFKS + FOLFOX4	FOLFOX4	15 mL Bid	(1–14 days) × 4	①②⑥
Tie-bin et al^[49]^	43/25	41/27	Ⅱ–Ⅳ	Rectal	61.4 ± 12.3	61.8 ± 12.6	FFKS + FOLFOX4	FOLFOX4	12 mL Qd	(1–15 days) × 4	①②③④⑤
Zhihua^[50]^	24/19	25/18	Ⅳ	Colorectal	54.59 ± 7.96	54.85 ± 7.94	FFKS + FOLFOX4	FOLFOX4	40 mL Qd	15 days	①②③④⑤
Qing et al^[51]^	27/14	26/15	Advanced	Rectal	55. 1 ± 6. 8	53. 6 ± 6. 1	FFKS + FOLFOX4	FOLFOX4	20 mL Qd	(1–14 days) × 4	②③④⑤

Outcome indicators: ① objective response rate; ② disease control rate; ③ quality of life; ④ white blood cell count; ⑤ nausea and vomiting; and ⑥ immune cell improvement.

AD = Aidi injection, Bid = twice daily, C = chemotherapy group, CapeOX = carboplatin + pemetrexed, FFBM = compound cantharidin capsules, FFKS = Compound Sophora flavescens injection, FOLFIRI = 5-fluorouracil + calcium folinate + irinotecan + cisplatin, FOLFOX = 5-fluorouracil + oxaliplatin + calcium folinate, HCS = Huachansu capsules, KA = Kangai injection, KAZS = kangai Injection, KLT = Kanglaite, mFOLFOX6: oxaliplatin + leucovorin + fluorouracil, Qd = once daily, T = proprietary Chinese medicines combined with chemotherapy group (test group), Tid = three times daily, TLBZ = Tenglong Buzhong Tang, TP = paclitaxel + cisplatin, XAP= Xiaoaiping, XELOX = oxaliplatin + capecitabine, YDZ = Brucea javanica oil emulsion injection, YZXJ = Yangzheng Xiaoji capsules.

### 3.2. Evaluation of literature quality

**Figure 2.**
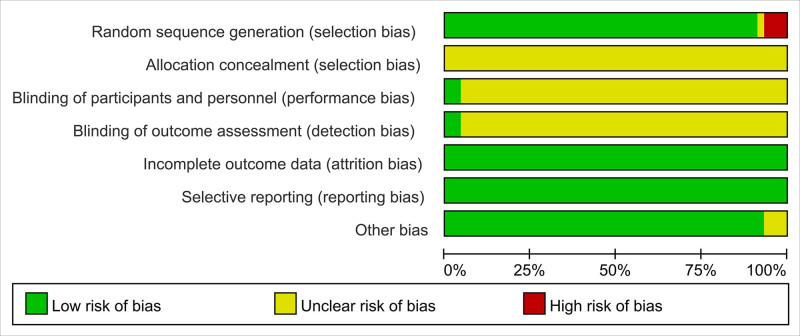
Proportion of projects with deviation risk generated by incorporating literature.

All 45 articles included were Chinese RCTs, and the intergroup data were comparable. As for the random method, 12 items^[21,23,33,40–43,46,48,49,51]^ were randomly assigned using the random number table method, with 1 item^[34]^ in the order of visit, 2 items^[24,35]^ in the treatment method, 1 item^[36]^ rated as “low risk” using the envelope method, 2 items^[9,32]^ using dynamic randomization, 2 items^[8,13]^ using random drawing, and 1 study^[18]^ explicitly using a randomized, controlled, and single blind approach. None of the 45 studies explained the hidden allocation method, rated as “clear risk.” Forty-three items were rated as “unclear risk” without specifying whether blind methods were used. All 43 studies did not indicate the implementation of blind methods for outcome evaluation and were rated as “clear risk.” In terms of selective reporting of research results, 45 studies were rated as “low risk.” However, none of these studies provided explanations for any other biases and were therefore rated as having a “clear risk.” The evaluation of literature quality is shown in Figure 2.

### 3.3. Evidence network

**Figure 3.**
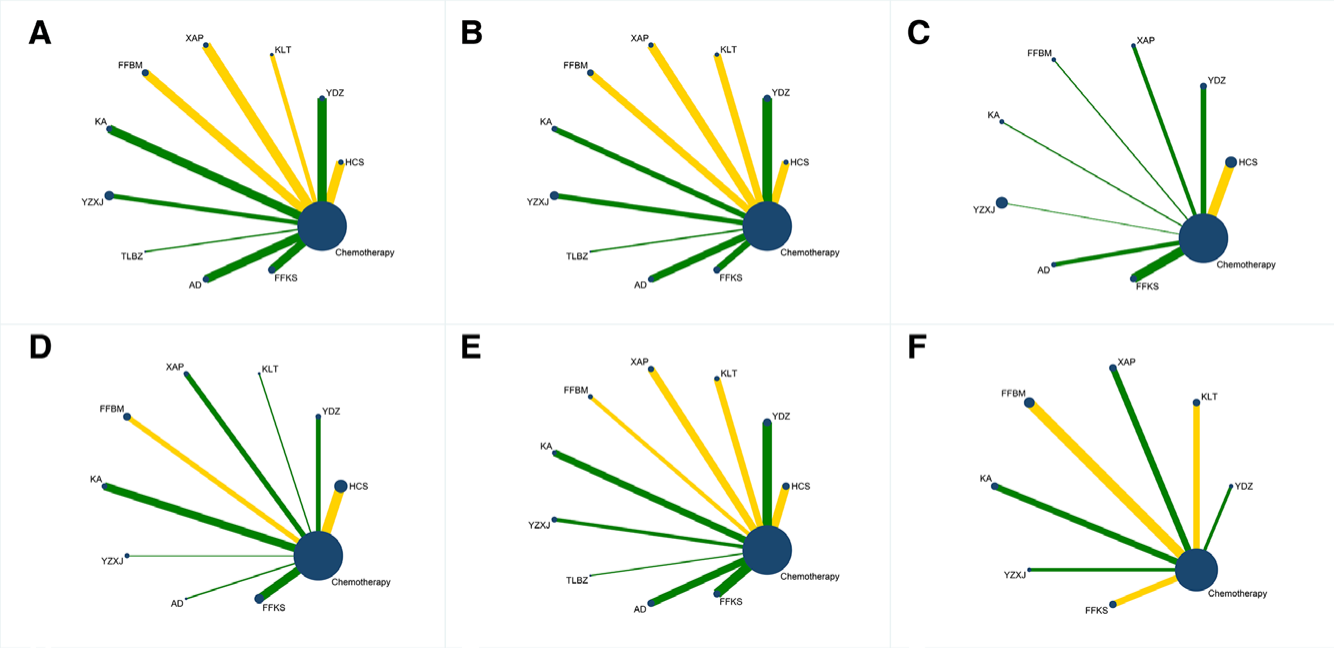
Evidence network for each outcome indicator. (A) DCR, (B) ORR, (C) QOL, (D) leukopenia, (E) nausea and vomiting, (F) CD4+/CD8+. DCR = disease control rate, ORR = objective response rate, QOL = quality of life.

A network evidence plot was generated in which each node represents an intervention and its size reflects the number of randomized participants exposed to that intervention; the thickness of each edge connecting two nodes is proportional to the number of randomized controlled trials directly comparing the two interventions. The plots for each outcome indicator in the evidence do not have a closed loop, as shown in Figure 3.

### 3.4. Mesh meta-analysis league table

#### 3.4.1. The DCR for different treatments

**Figure 4.**
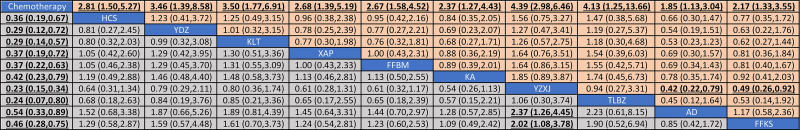
DCR league table. DCR = disease control rate.

DCR for different treatment was as follows: Huachansu capsule + chemotherapy (OR = 2.81, 95% CI: 1.50–5.27), Brucea javanica oil emulsion injection + chemotherapy (OR = 3.46, 95% CI: 1.39–8.58), Kanglaite + chemotherapy (OR = 3.50, 95% CI: 1.77–6.91), Xiaoaiping + chemotherapy (OR = 2.68, 95% CI: 1.39–5.19), compound cantharidin capsule + chemotherapy (OR = 2.67, 95% CI: 1.58–4.52), Kangai injection + chemotherapy (OR = 2.37, 95% CI: 1.27–4.43), Yangzheng Xiaoji capsules + chemotherapy (OR = 4.39, 95% CI: 2.98–6.46), Tenglong Buzhong Tang + chemotherapy (OR = 4.13, 95% CI: 1.25–13.66), Aidi injection + chemotherapy (OR = 1.85, 95% CI: 1.13–3.04), and compound Sophora flavescens injection + chemotherapy (OR = 2.17, 95% CI: 1.33–3.55). Among these, Yangzheng Xiaoji capsules + chemotherapy showed better efficacy than Aidi injection + chemotherapy (OR = 2.37, 95% CI: 1.26–4.45) and compound Sophora flavescens injection + chemotherapy (OR = 2.02, 95% CI: 1.08–3.78), with statistical significance (*P* < .05), as shown in Figure 4.

#### 3.4.2. Objective response rate (ORR)

**Figure 5.**
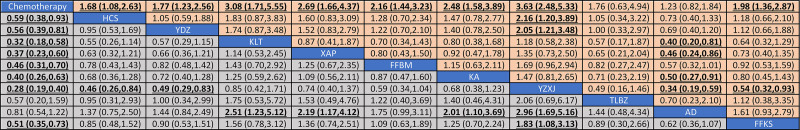
ORR league table. ORR = objective response rate.

Huachansu capsule + chemotherapy (OR = 1.68, 95% CI: 1.08–2.63), Brucea javanica oil emulsion injection + chemotherapy (OR = 1.77, 95% CI: 1.23–2.56), Kanglaite + chemotherapy (OR = 3.08, 95% CI: 1.71–5.55), Xiaoaiping + chemotherapy (OR = 2.69, 95% CI: 1.66–4.37), compound cantharidin capsule + chemotherapy (OR = 2.16, 95% CI: 1.44–3.23), Kangai injection + chemotherapy (OR = 2.48, 95% CI: 1.58–3.89), Yangzheng Xiaoji capsules + chemotherapy (OR = 3.63, 95% CI: 2.48–5.33), and compound Sophora flavescens injection + chemotherapy (OR = 1.98, 95% CI: 1.36–2.87), as adjuvant therapy, have better efficacy than simple chemotherapy. Among them, Yangzheng Xiaoji capsules + chemotherapy is superior to Huachansu capsules + chemotherapy (OR = 2.16, 95% CI: 1.20–3.89), Brucea javanica oil emulsion injection + chemotherapy (OR = 2.05, 95% CI: 1.21–3.48), and Aidi injection + chemotherapy (OR = 2.96, 95% CI: 1.69–5.16). Compound Sophora flavescens injection + chemotherapy (OR = 1.83, 95% CI: 1.08–3.13), Kanglaite + chemotherapy (OR = 2.51, 95% CI: 1.23–5.12), Xiaoaiping + chemotherapy (OR = 2.19, 95% CI: 1.17–4.12), and Kangai injection + chemotherapy (OR = 2.01, 95% CI: 1.10–3.69) have better therapeutic effects than Aidi injection + chemotherapy, with statistically significant differences (*P* < .05), as shown in Figure 5.

#### 3.4.3. Quality of life (QOL)

**Figure 6.**
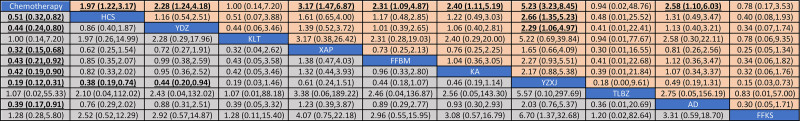
QOL league table. QOL = quality of life.

Huachansu capsules + chemotherapy (OR = 1.97, 95% CI: 1.22–3.17), Brucea javanica oil emulsion injection + chemotherapy (OR = 2.28, 95% CI: 1.24–4.18), Xiaoaiping + chemotherapy (OR = 3.17, 95% CI: 1.47–6.87), compound cantharidin capsules + chemotherapy (OR = 2.31, 95% CI: 1.09–4.87), Kangai injection + chemotherapy (OR = 2.40, 95% CI: 1.11–5.19), Yangzheng Xiaoji capsules + chemotherapy (OR = 5.23, 95% CI: 3.23–8.45), and Aidi injection + chemotherapy (OR = 2.58, 95% CI: 1.10, 6.03) have better therapeutic effect compared to simple chemotherapy. Among them, the combination of Yangzheng Xiaoji capsule and chemotherapy has shown a superior therapeutic effect compared with Huachansu capsule and chemotherapy (OR = 2.66, 95% CI: 1.35–5.23). Additionally, it has also demonstrated better results than Brucea Javanica oil emulsion injection and chemotherapy (OR = 2.29, 95% CI: 1.06–4.97). These differences are statistically significant (*P* < .05), as illustrated in Figure 6.

#### 3.4.4. Improvement of leukopenia

**Figure 7.**
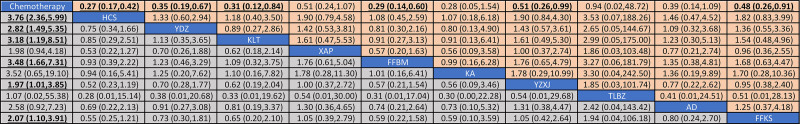
Leukopenia league table.

Huachansu capsule + chemotherapy (OR = 0.27, 95% CI: 0.17–0.42), Brucea javanica oil emulsion injection + chemotherapy (OR = 0.35, 95% CI: 0.19–0.67), compound cantharidin capsule + chemotherapy (OR = 0.29, 95% CI: 0.14–0.60), Yangzheng Xiaoji capsule + chemotherapy (OR = 0.51, 95% CI: 0.26–0.99), and compound Sophora flavescens injection + chemotherapy (OR = 0.48, 95% CI: 0.26–0.91) have been shown to significantly improve leukopenia compared with single purification. This combination therapy demonstrates a statistically significant difference (*P* < .05), as depicted in Figure 7.

#### 3.4.5. Improvement of nausea and vomiting compared with single purification when combined with chemotherapy

**Figure 8.**
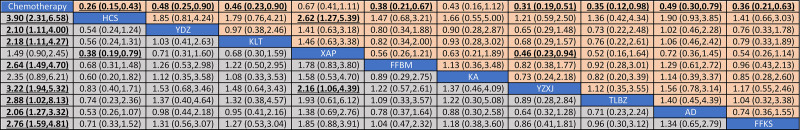
Nausea and vomiting league table.

Huachansu capsules + chemotherapy (OR = 0.26, 95% CI: 0.15–0.43), Brucea javanica oil emulsion injection + chemotherapy (OR = 0.48, 95% CI: 0.25–0.90), Kanglaite + chemotherapy (OR = 0.46, 95% CI: 0.23–0.90), compound cantharidin capsules + chemotherapy (OR = 0.38, 95% CI: 0.21–0.67), Yangzheng Xiaoji capsules + chemotherapy (OR = 0.31, 95% CI: 0.19–0.51), Tenglong Buzhong Tang + chemotherapy (OR = 0.35, 95% CI: 0.12–0.98), Aidi injection combined with chemotherapy (OR = 0.49, 95% CI: 0.30–0.79), and compound Sophora flavescens injection combined with chemotherapy (OR = 0.36, 95% CI: 0.21–0.63). These findings were statistically significant (*P* < .05), as shown in Figure 8.

#### 3.4.6. Improvement of immune function

**Figure 9.**
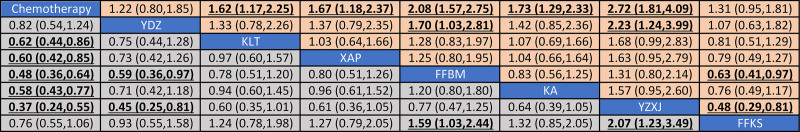
CD4+/CD8+ league table.

Kanglaite + chemotherapy (OR = 1.62, 95% CI: 1.17–2.25), Xiaoaiping + chemotherapy (OR = 1.67, 95% CI: 1.18–2.37), compound cantharidin capsule + chemotherapy (OR = 2.08, 95% CI: 1.57–2.75), Kangai injection + chemotherapy (OR = 1.73, 95% CI: 1.29–2.33), and Yangzheng Xiaoji capsule + chemotherapy (OR = 2.72, 95% CI: 1.81–4.09) are superior to chemotherapy alone. The combination of compound cantharidin capsule and chemotherapy is better than that of Brucea javanica oil emulsion injection + chemotherapy (OR = 1.70, 95% CI: 1.03–2.81) and compound Sophora flavescens injection + chemotherapy (OR = 1.59, 95% CI: 1.03–2.44), and Yangzheng Xiaoji capsule + chemotherapy is better than that of Brucea javanica oil emulsion injection + chemotherapy (OR = 2.23, 95% CI: 1.24–3.99) and compound Sophora flavescens injection + chemotherapy (OR = 2.07, 95% CI: 1.23–3.49), with statistically significant differences (*P* < .05), as shown in Figure 9.

### 3.5. SUCRA sorting

**Figure 10.**
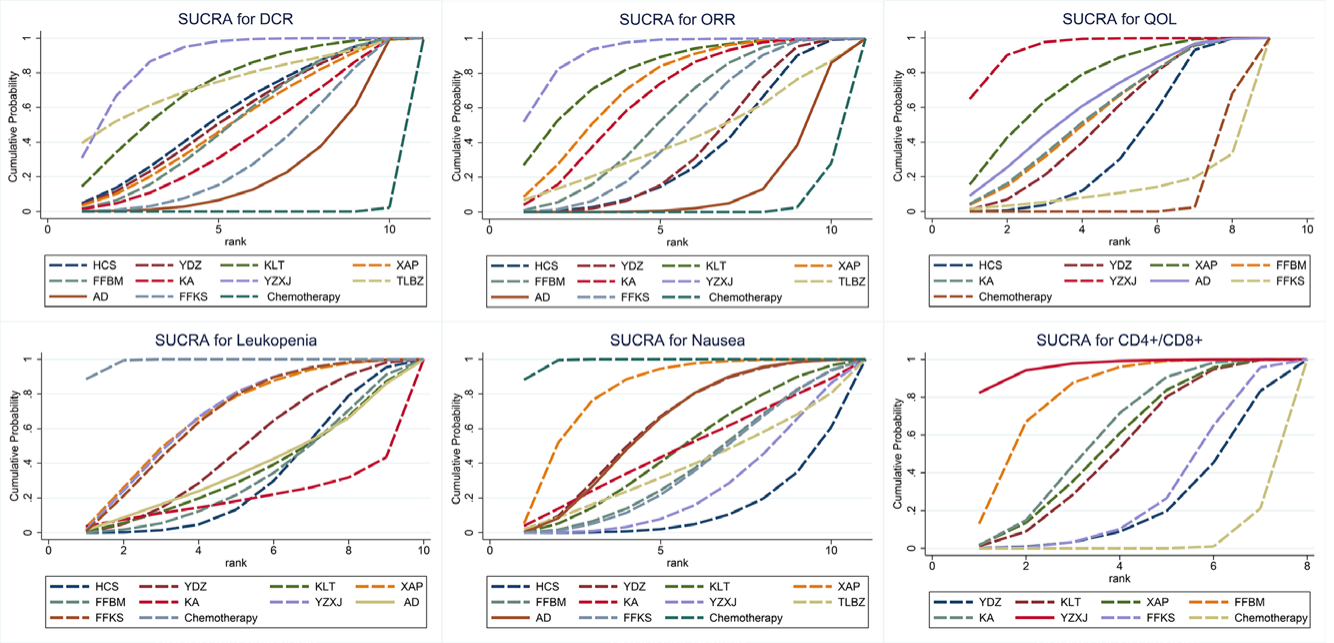
Cumulative sorting chart SUCRA. SUCRA = surface under the cumulative ranking.

The SUCRA rankings were used to evaluate the effectiveness of 10 traditional Chinese patent medicines and simple preparations in treating colorectal cancer. The results of the reticular meta-analysis showed that the main outcome indicators were as follows: In terms of improving the ORR of colorectal cancer, the ranking of traditional Chinese patent medicines and simple preparations is as follows: Yangzheng Xiaoji capsule (SUCRA = 92.7%) > Kanglaite (SUCRA = 80.9%) > Xiaoaiping (SUCRA = 73.1%) > Kangai injection (SUCRA = 66.9%) > compound cantharides capsule (SUCRA = 55.6%) > compound Sophora flavescens injection (SUCRA = 47.8%) > Tenglong Buzhong Decoction (SUCRA = 42.0%) > Brucea Javanica Oil Emulsion injection (SUCRA = 38.4%) > cinobufagin capsules (SUCRA = 35.1%) > Aidi injection (SUCRA = 14.5%) > chemotherapy (SUCRA = 3.1%). In terms of improving the DCR, Traditional Chinese patent medicines and simple preparations ranked as Yangzheng Xiaoji capsule (SUCRA = 85.8%) > Tenglong Buzhong Decoction (SUCRA = 73.4%) > Kanglaite (SUCRA = 68.7%) > Brucea javanica oil emulsion injection (SUCRA = 66.2%) > cinobufagin capsule (SUCRA = 54.4%) > Xiaoaiping (SUCRA = 50.8%) > compound cantharides capsule (SUCRA = 50.3%) > Kangai injection (SUCRA = 41.7%) > compound Sophora flavescens injection (SUCRA = 34.3%) > Aidi injection (SUCRA = 24.1%) > chemotherapy (SUCRA = 0.3%). In improving the QOL of colorectal cancer patients, the ranking of traditional Chinese patent medicines and simple preparations is Yangzheng Xiaoji capsule (SUCRA = 94.0%) > Xiaoaiping (SUCRA = 72.9%) > Aidi injection (SUCRA = 62.6%) > Kangai injection (SUCRA = 59.1%) > compound cantharides capsule (SUCRA = 57.4%) > Brucea javanica oil emulsion injection (SUCRA = 55.8%) > cinobufagin capsule (SUCRA = 47.5%) > Tenglong Buzhong Decoction (SUCRA = 37.0%) > Kanglaite (SUCRA = 28.5%) > compound Kushen injection (SUCRA = 18.2%) > chemotherapy (SUCRA = 16.9%). In improving the decrease in WBC in colorectal cancer patients after chemotherapy, traditional Chinese patent medicines and simple preparations ranked as Huachansu capsule (SUCRA = 78.4%) > compound cantharides capsule (SUCRA = 72.1%) > Kangai injection (SUCRA = 65.7%) > Kanglaite (SUCRA = 62.5%) > Brucea javanica oil emulsion injection (SUCRA = 57.4%) > Aidi injection (SUCRA = 54.4%) > compound Sophora flavescens injection (SUCRA = 41.1%) > Xiaoaiping (SUCRA = 39.0%) > Yangzheng Xiaoji capsule (SUCRA = 38.6%) > Tenglong Buzhong decoction (SUCRA = 33.9%) > chemotherapy (SUCRA = 6.9%). In terms of improving the incidence of nausea and vomiting in colorectal cancer patients after chemotherapy, traditional Chinese patent medicines and simple preparations ranked as cinobufagin capsule (SUCRA = 86.6%) > Yangzheng Xiaoji capsule (SUCRA = 75.1%) > Tenglong Buzhong decoction (SUCRA = 63.1%) > compound Sophora flavescens injection (SUCRA = 62.5%) > compound cantharides capsule (SUCRA = 59.9%) > Kangai injection (SUCRA = 53.6%) > Aidi injection (SUCRA = 39.8%) > Brucea javanica oil emulsion injection (SUCRA = 38.2%) > Xiaoaiping (SUCRA = 19.1%) > chemotherapy (SUCRA = 1.4%). In terms of improving immune function, traditional Chinese patent medicines and simple preparations ranked as Yangzheng Xiaoji capsule (SUCRA = 96.5%) > compound cantharis capsule (SUCRA = 80.2%) > Kangai injection (SUCRA = 60.1%) > Xiaoaiping (SUCRA = 55.8%) > Kanglaite (SUCRA = 52.2%) > compound Sophora flavescens injection (SUCRA = 29.1%) > Brucea javanica oil emulsion injection (SUCRA = 22.8%) > chemotherapy (SUCRA = 3.2%), as shown in Figure 10.

### 3.6. Inconsistency inspection

As none of the 6 outcome indicators in this study resulted in a closed loop, a consistency test was not conducted.

### 3.7. Publication bias test

**Figure 11.**
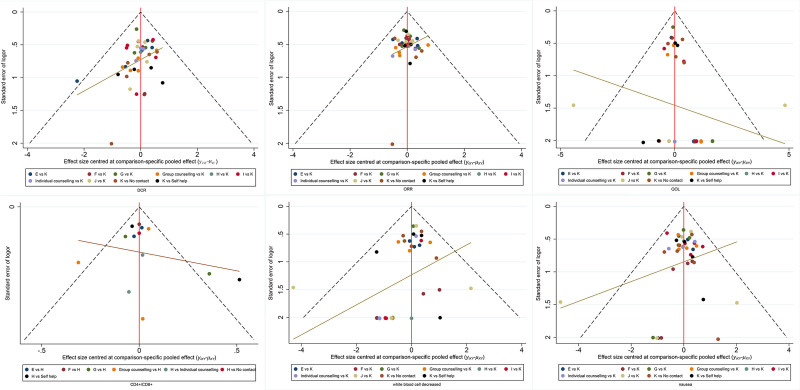
Publication bias.

A corrected comparison funnel plot was drawn for the main outcome measure for publication bias testing, and the results showed good symmetry without significant publication bias (Fig. 11).

## 4. Discussion

The early symptoms of colorectal cancer are atypical, which often leads to patients being diagnosed in the middle to advanced stage, causing them to miss the best surgical opportunity. Therefore, chemotherapy is often used in clinical treatment. However, chemotherapy drugs can damage normal cells such as hematopoietic cells, immune cells, and nerve cells while killing cancer cells. Therefore, common adverse reactions of chemotherapy include bone marrow suppression, peripheral neurotoxicity, decreased levels of various blood cells, compromised immune function, gastrointestinal reactions, and liver and kidney damage, among others. TCM categorizes colorectal cancer into classifications such as “intestinal wind,” “intestinal qi,” “visceral toxin,” and “intestinal tumor.” The “Internal Classic” states: For those with intestinal qi, cold qi is present outside the intestines, competing with Wei Qi, and Qi cannot be stimulated. There is also the saying in the book “Lingshu”: If there is a knot, the Qi will return, the Wei Qi will remain, and it cannot be reversed. If body fluid remains in the body for an extended period, it can combine and form intestinal tumors. It is pointed out that intestinal tumors and intestinal qi can be caused by the lack of Wei Qi and the invasion of evil energy. TCM believes that the development of colorectal cancer is closely linked to emotional internal injury and improper diet. It is believed that there is a deficiency of proper qi in the body, allowing external pathogens to enter and accumulate in the large intestine veins. Over time, this accumulation leads to the onset of the disease. According to the “Medical Zong Jin Jian: Accumulation,” it is recorded that “repeated attacks and supplements are necessary to maintain a stable period.” It is pointed out that individuals who have an accumulation in their diseased intestines may experience abdominal lumps, and the root cause of this condition lies in blood circulation. The spleen plays a crucial role in regulating qi and blood biochemistry. Thus, this disease is primarily attributed to spleen deficiency. If there is spleen deficiency, hydration becomes disadvantageous, and there is an imbalance between yin and yang. Dampness accumulates in the middle energizer and condenses into phlegm and yin. Blood stasis persists in the meridians and becomes cancerous and toxic over time. Therefore, the treatment principle should be to strengthen the spleen, promote dampness, detoxify, and remove stasis. At present, numerous studies have demonstrated that the utilization of traditional Chinese patent medicines and simple preparations in the treatment of colorectal cancer during radiotherapy and chemotherapy can not only mitigate the adverse effects of these treatments on patients but also significantly improve the therapeutic outcomes. How to select suitable drugs from a variety of traditional Chinese patent medicines and simple preparations has always been a topic of concern.

This study comprehensively evaluated 10 kinds of traditional Chinese patent medicines and simple preparations commonly used in the clinical treatment of advanced colorectal cancer from 6 outcome indicators: short-term efficacy (ORR and DCR), QOL, WBC, immune function improvement, nausea, and vomiting. The analysis of this study demonstrates that various TCM combined chemotherapy regimens significantly improve DCR compared with chemotherapy alone. Specifically, Huachansu capsule combined with chemotherapy (OR = 2.81, 95% CI: 1.50–5.27), Brucea javanica oil emulsion injection combined with chemotherapy (OR = 3.46, 95% CI: 1.39–8.58), and Kanglaite injection combined with chemotherapy (OR = 3.50, 95% CI: 1.77–6.91) all show higher DCRs. Among them, the combination of Yangzheng Xiaoji capsules with chemotherapy (OR = 4.39, 95% CI: 2.98–6.46) and Tenglong Buzhong Tang with chemotherapy (OR = 4.13, 95% CI: 1.25–13.66) are particularly notable for their efficacy. These results suggest that TCM combined with chemotherapy may have potential advantages in improving DCRs. In terms of ORR, our analysis also found that TCM combined chemotherapy regimens have better efficacy than chemotherapy alone. For instance, the combination of Yangzheng Xiaoji capsules with chemotherapy (OR = 3.63, 95% CI: 2.48–5.33) is superior in improving the ORR compared with Huachansu capsules with chemotherapy (OR = 2.16, 95% CI: 1.20–3.89) and Brucea javanica oil emulsion injection with chemotherapy (OR = 2.05, 95% CI: 1.21–3.48), with statistically significant differences (*P* < .05). These results further confirm the potential value of TCM combined with chemotherapy in improving the ORR in patients with advanced colorectal cancer. Improvement in QOL is one of the important indicators for evaluating the efficacy of treatment regimens. This study’s analysis shows that Huachansu capsules combined with chemotherapy (OR = 1.97, 95% CI: 1.22–3.17) and Yangzheng Xiaoji capsules combined with chemotherapy (OR = 5.23, 95% CI: 3.23–8.45) are superior in improving QOL compared with chemotherapy alone. Particularly, the combination of Yangzheng Xiaoji capsules with chemotherapy, compared with Huachansu capsules with chemotherapy (OR = 2.66, 95% CI 1.35–5.23) and Brucea javanica oil emulsion injection with chemotherapy (OR = 2.29, 95% CI 1.06–4.97), shows statistically significant differences (*P* < .05). This indicates that TCM combined with chemotherapy regimens may play a significant role in improving the QOL for patients with advanced colorectal cancer. In terms of improving leukopenia, our analysis found that Huachansu capsules combined with chemotherapy (OR = 0.27, 95% CI: 0.17–0.42) and Brucea javanica oil emulsion injection combined with chemotherapy (OR = 0.35, 95% CI: 0.19–0.67) significantly improve chemotherapy-induced leukopenia, with statistically significant differences compared with chemotherapy alone (*P* < .05). This suggests that these TCM combined chemotherapy regimens may help alleviate chemotherapy-related hematological toxicities. Nausea and vomiting are common side effects of chemotherapy. Our analysis shows that Huachansu capsules combined with chemotherapy (OR = 0.26, 95% CI: 0.15–0.43) and Brucea javanica oil emulsion injection combined with chemotherapy (OR = 0.48, 95% CI 0.25–0.90) significantly improve chemotherapy-induced nausea and vomiting, with statistically significant differences compared with chemotherapy alone (*P* < .05). This indicates that TCM combined with chemotherapy regimens may help alleviate chemotherapy-related gastrointestinal toxicities. Improvement in immune function is crucial for the prognosis of patients with advanced colorectal cancer. Our analysis found that Kanglaite injection combined with chemotherapy (OR = 1.62, 95% CI: 1.17–2.25) and Yangzheng Xiaoji capsules combined with chemotherapy (OR = 2.72, 95% CI: 1.81–4.09) are superior in improving immune function compared with chemotherapy alone. Particularly, the combination of Yangzheng Xiaoji capsules with chemotherapy, compared with Brucea javanica oil emulsion injection with chemotherapy (OR = 2.23, 95% CI: 1.24–3.99) and compound Sophora flavescens injection with chemotherapy (OR = 2.07, 95% CI: 1.23–3.49), shows statistically significant differences (*P* < .05). This suggests that TCM combined with chemotherapy regimens may play a significant role in improving immune function in patients with advanced colorectal cancer.

The article also ranked the efficacy of the 10 TCMs included in the study. In terms of improving ORR and DCR, Yangzheng Xiaoji capsule has the best effect (ORR: SUCRA = 92.7%, DCR: SUCRA = 85.8%). Yangzheng Xiaoji capsule is mainly composed of TCM, such as Ligustrum lucidum, Ganoderma lucidum, and ginseng. Huangqi is a vital medicine for tonifying qi, among others. It can significantly improve the immune system and alleviate physical weakness in cancer patients.^[52]^ It plays a role in supplementing qi, nourishing yin, and strengthening the body. It can also inhibit the proliferation of tumor cells by regulating certain specific signaling pathways.^[53]^ The saponins in ginseng can inhibit the invasion and metastasis of tumor cells and enhance the body’s immunity.^[54]^ Multiple chemical components in Hedyotis diffusa can induce cancer cell apoptosis.^[55]^ Xu Changqing contains various pharmacologically active ingredients that regulate immunity and fight against tumors.^[56]^ The triterpenoid compounds in Ganoderma lucidum also have significant in vitro cytotoxic effects, which can induce tumor cell differentiation.^[57]^ In addition, the combination of Huangqi and Zedoary Turmeric is a representative drug of the method of tonifying qi and promoting blood circulation, which has the effect of inhibiting tumor metastasis.^[58]^ On the basis of strengthening the spleen and kidney, the entire formula has the effects of dispersing blockages, clearing channels, detoxifying, and anticancer. This highlights the treatment principle of “supporting the right without retaining evil, eliminating evil without damaging the right.” Experiments have shown that Yangzheng Xiaoji capsule can significantly interfere with the phosphoinositol 3-kinase/protein kinase B signaling pathway, which plays a crucial role in the invasion and metastasis of tumor cells. Blocking this pathway means inhibiting the adhesion and migration of tumor cells, thereby effectively controlling the development of tumor lesions.^[59]^ The 15 RCTs included by Lishuang et al^[60]^ showed that compared with simple chemotherapy, the combination of Yangzheng Xiaoji capsule and chemotherapy can improve the clinical efficacy of gastrointestinal malignant tumors, improve patients’ QOL, regulate immune function, and reduce adverse reactions caused by chemotherapy. Yangzheng Xiaoji capsules contain a variety of active ingredients that have antitumor and immune regulatory properties. These ingredients have a significant impact on inhibiting the metastasis, invasion, and in vivo growth of colorectal cancer. With the change of medical models, QOL indicators have become one of the important evaluation indicators for evaluating the clinical treatment effectiveness of cancer patients, especially those in the middle and advanced stages. In this study, 7 kinds of traditional Chinese patent medicines and simple preparations can improve QOL of patients with advanced colorectal cancer after chemotherapy (*P* < .05). Compared with simple chemotherapy, Huachansu capsule, Brucea javanica oil emulsion injection, Xiaoaiping, compound cantharides capsule, Kangai injection, Yangzheng Xiaoji capsule, and Aidi injection have better efficacy, of which Yangzheng Xiaoji capsule has the best efficacy (SUCRA = 94.0%). In addition, Yangzheng Xiaoji capsule also shows great advantages in improving the immune function of patients (SUCRA = 96.5%).

In terms of reducing the incidence of leukopenia and nausea and vomiting, Huachansu capsules (white blood cells: SUCRA = 78.4%, nausea and vomiting: SUCRA = 86.6%) are the optimal choice. Huachansu is a widely used antitumor TCM preparation in clinical practice. It is an extract of dried toad skin and has the effects of clearing heat, detoxifying, promoting diuresis, reducing swelling, and softening and dispersing nodules. The main active ingredients of Huachansu include toad venom ligands, peptides, indole alkaloids, etc.^[61]^ The “Shennong Materia Medica Classic” once recorded that “toads taste pungent and cold, dominate evil energy, alleviate symptoms, promote blood circulation, treat carbuncles and yin sores, and can be taken without causing fever.” This provides a theoretical basis for the clinical treatment of tumors with Huachansu.^[62]^ In recent years, clinical studies have found that compared with conventional chemotherapy drugs, cinobufagin, either alone or in combination, offers several advantages. These include fewer toxic side effects, reduced adverse reactions, and increased anticancer activity. It can also improve the patient’s immune system and enhance their QOL.^[63,64]^ Jinpeng et al^[10]^ randomly divided 96 patients with colon cancer into 2 groups. The control group consisted of 48 cases who received chemotherapy with the capecitabine + oxaliplatin regimen. In addition to the control group, the treatment group included 48 cases who were treated with Huachansu capsules. Both groups had one cycle of 3 weeks, with 2 cycles of treatment. Result: The improvement rate of QOL in the treatment group was 87.5%, while the improvement rate of QOL in the control group was 68.8%. The improvement of QOL in the treatment group was better than that in the control group (*P* < .01). Chemotherapy not only controls tumor development but also causes a series of adverse reactions, such as bone marrow suppression, inflammatory reactions, gastrointestinal reactions, and neurotoxicity, which makes patients unable to tolerate chemotherapy and limits the effectiveness of chemotherapy. Therefore, there is an urgent need to find treatment plans to reduce the occurrence of adverse reactions in patients after chemotherapy. Yun et al^[65]^ randomly divided 71 patients with advanced intestinal tumors into 2 groups. Thirty-three cases in the control group were treated with the cisplatin + 5-fluorouracil regimen chemotherapy, while 38 cases in the treatment group received a combination therapy of cinobufagin injection in addition to the control group’s treatment. Both groups received one course of treatment after 21 days, consisting of 3 treatment sessions. Result: The incidence of gastrointestinal reactions and bone marrow suppression reactions in the treatment group was lower than that in the control group (*P* < .05), and the proportion of grade III and IV adverse reactions was lower than that in the control group (*P* < .05). This indicates that the combination of Huachansu injection and chemotherapy can significantly reduce the toxic side effects of bone marrow suppression and other chemotherapy treatments. Huachansu has been widely used in the treatment of tumors such as liver cancer, gastric cancer, lung cancer, and colon cancer. The results of this study show that traditional Chinese patent medicines and simple preparations can improve the clinical efficacy of patients with advanced colorectal cancer, reduce the adverse reactions caused by chemotherapy, and have the effect of enhancing efficacy and reduce toxicity.

This study still has the following limitations: The quality of the literature included in the study is relatively low, with only one out of the 45 studies explicitly indicating the use of blinding methods, and none of the studies have reported the allocation of concealed and outcome evaluator blinding methods. The evaluation of literature quality is unclear, and there is a certain risk of publication bias. Additionally, the number of included studies and sample size are limited. It is worth noting that Tenglong Buzhong capsule is only included in one study, which may lead to an exaggeration of the efficacy of its combination chemotherapy for colorectal cancer. Moreover, the study included in this analysis is based on a small sample size, which reduces the statistical reliability of the findings. Additionally, the lack of research and comparison of different traditional Chinese patent medicines and simple preparations affects the stability of the analysis results. Furthermore, the observation period of the included studies is relatively short, with most focusing on 2 to 3 months of treatment, thus lacking long-term evaluation of efficacy and safety.

To summarize, current evidence suggests that the effectiveness of combining traditional Chinese patent medicines and simple preparations with conventional chemotherapy is often superior to conventional chemotherapy alone in treating advanced colorectal cancer. This combination can also reduce the occurrence of adverse reactions and enhance efficacy while reducing toxicity. This study aims to provide evidence for rational drug use in clinical practice, where personalized medication plans can be developed based on specific disease symptoms and tailored to the individual needs of patients. However, this study is limited by the quality and quantity of included studies, and there is a significant variation in the efficacy ranking of each traditional Chinese patent medicine and simple preparation outcome indicator. These conclusions need to be further verified by larger sample sizes, high-quality reports, and standardized methodologies.

## 5. Conclusion

The results showed that all 10 types of traditional Chinese patent medicines and simple preparations were able to improve the DCR, ORR, and QOL in patients with advanced colorectal cancer. Additionally, these medicines were found to enhance the immune function of patients and reduce the occurrence of adverse reactions.

## Author contributions

**Conceptualization:** Mingxing Wang, Qingming Sun.

**Investigation:** Mingxing Wang.

**Methodology:** Mingxing Wang, Gongyi Wu.

**Project administration:** Mingxing Wang, Wanhui Dong.

**Writing – original draft:** Mingxing Wang, Qingming Sun.

**Writing – review & editing:** Mingxing Wang.

**Resources:** Wanhui Dong.

**Software:** Wanhui Dong.

**Validation:** Qingming Sun, Gongyi Wu.

**Visualization:** Gongyi Wu.
